# Global Diversity of Ascidiacea

**DOI:** 10.1371/journal.pone.0020657

**Published:** 2011-06-20

**Authors:** Noa Shenkar, Billie J. Swalla

**Affiliations:** Department of Biology, University of Washington, Seattle, Washington, United States of America; University of Washington Friday Harbor Labs, Friday Harbor, Washington, United States of America; Institute of Marine Research, Norway

## Abstract

The class Ascidiacea presents fundamental opportunities for research in the fields of development, evolution, ecology, natural products and more. This review provides a comprehensive overview of the current knowledge regarding the global biodiversity of the class Ascidiacea, focusing in their taxonomy, main regions of biodiversity, and distribution patterns. Based on analysis of the literature and the species registered in the online World Register of Marine Species, we assembled a list of 2815 described species. The highest number of species and families is found in the order Aplousobranchia. Didemnidae and Styelidae families have the highest number of species with more than 500 within each group. Sixty percent of described species are colonial. Species richness is highest in tropical regions, where colonial species predominate. In higher latitudes solitary species gradually contribute more to the total species richness. We emphasize the strong association between species richness and sampling efforts, and discuss the risks of invasive species. Our inventory is certainly incomplete as the ascidian fauna in many areas around the world is relatively poorly known, and many new species continue to be discovered and described each year.

## Introduction

Ascidians (Phylum Chordata, Class Ascidiacea), or sea squirts, are the largest and most diverse class of the sub-phylum Tunicata (also known as Urochordata). They comprise approximately 3000 described species found in all marine habitats from shallow water to the deep sea [1]–[3]. The group was initially difficult for zoologists to classify systematically, although ascidians were recognized as a distinct group as early as Aristotle [1]. The first clear description of an ascidian was made by Schlosser in 1756 in a letter entitled “An account of a curious, fleshy, coral-like substance”. This specimen was dredged along the British Islands and was actually what we know now as the widely distributed colonial ascidian *Botryllus schlosseri* [4].

The name “tunicate” (sub-phylum *Tunicata*) was first coined by Lamarck [5] for ascidians, pyrosomes, and salps [6], [7]. The name originates from the polysaccharide-containing tunic that envelops the animal and forms a somewhat flexible skeleton [1]. Milne Edwards [8] mistakenly included the Bryozoa in this group, and both, together with the Brachiopoda, were included in the Mollusca by Hancock [9]. Savigny [10] also recognized the Tunicata (ascidians, salps, doliolids, and appendicularians) as a distinct group separate from the Mollusca [6]. Finally, the chordate nature of the ascidian tadpole larva was recognized by Kowalevsky [11], and they were reclassified with chordates [12]. The name Urochordata was not used until Balfour [13] created it as a replacement name for Tunicata, presumably to emphasize the chordate affinity. Indeed, recent phylogenomic studies place the tunicates as the sister group to the vertebrates [14]–[16], suggesting that they are our closest relatives among the invertebrates, which provides a fertile ground for evolutionary and developmental studies [17].

**Figure 1:**
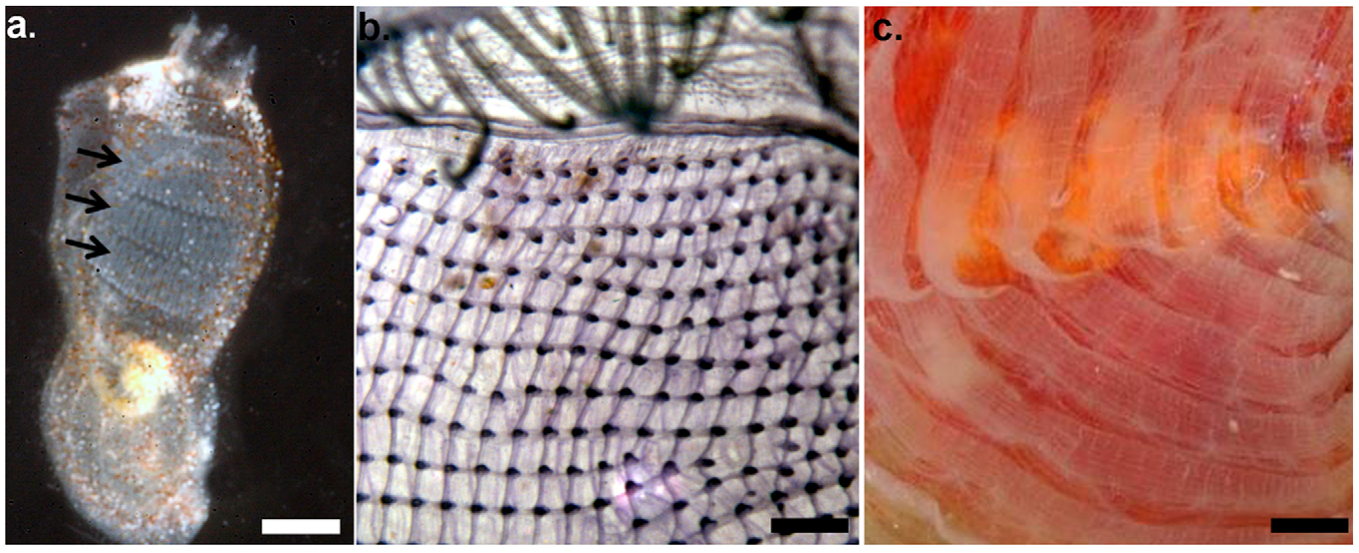
Ascidian branchial sac structure, a distinguishing taxonomic character. a) A simple branchial arrangement in an aplousobranch (*Didemnum* sp.). Arrows pointing out the straight stigmata rows. Photo: A. Shoob. Scale bar 1 mm; b) phlebobranch (*Ascidia* sp.) with longitudinal blood vessels; c) stolidobranch (*Herdmania momus*) with branchial folds. Photos: N Shenkar. Scale bar 1 mm and 4 mm respectively.

Following the original classification of Lahille [18], the class Ascidiacea is now divided into three orders based on the structure of the adult branchial sac: Aplousobranchia (simple), Phlebobranchia (vascular) and Stolidobranchia (folded) (Fig. 1). This is the current classification used by most ascidian taxonomists that also corresponds to molecular phylogenetic analysis based on the 18S rDNA [7], [19] as opposed to Perrier's [20] division that was based upon the position of the gonads and other morphological considerations and comprised only two orders: Enterogona and PleurogonaAscidians belonging to the order Aplousobranchia are all colonial while the Phlebobranchia and Stolidobranchia include both colonial and solitary species [7].

Adult ascidians are sessile, inhabiting a wide variety of habitats such as soft sediments, coral reefs and rocky substrates. They successfully foul various artificial substrata such as jetties, ship hulls, floating docks and other man-made structures all over the world [21], [22]. They remain sessile following larval settlement throughout their adult life, so they cannot avoid salinity or temperature changes, and thus larval behavior is critical [23], [24]. Only a few species can survive in salinities below 20–25‰ [22], [25], or above 44‰ [26], (Shenkar N. unpublished results). The tropical *Ecteinascidia thurstoni* has been recorded along the Suez Canal in habitats with salinity reaching 46‰ overgrowing metal pilings of jetties [26], while several species inhabit marine lakes in Indonesia with salinity of 28.5‰ [27]. In salinities below 22‰ larval development is severely affected [24], as is the health of adult zooids [28]. Nonetheless, highly tolerant species such as *Ciona intestinalis* survive a wide range of salinities (12–40‰), and can withstand short periods of lower salinities (<11‰) [29], [30]. In general, ascidians exhibit a wider tolerance to temperature range than salinities [22], [31]. Antarctic species can tolerate temperature as low as -1.9°C [32], while others can survive seawater temperature higher than 35°C in the Arabian Gulf [33].

Both salinity and temperature are among the most important environmental variables influencing ascidian recruitment and reproduction [31], [34]–[36]. Other factors that may affect spatial distribution and recruitment include light, substrate type, hydrodynamics, predation and competition [22], [34], [37]. Understanding the role of these factors in ascidian recruitment, dispersal and survival is crucial to our understanding of ascidian global distribution patterns.

Ascidians are a key ecological group because of their invasive potential and ability to thrive in eutrophic (nutrient-rich) environments. Introductions of non-indigenous ascidians into harbors in both tropical and temperate waters are now commonplace, with the rate of introductions increasing, sometimes creating severe damage to natural fauna by overgrowth [1], [22], [38]–[41] (reviewed in a special issue of Aquatic Invasions January 2009). For example, the solitary ascidians *Styela clava* and *Ciona intestinalis* have had an adverse effect on aquaculture along Canada's east coast, mainly on mussel culture [42]–[44]. *S. clava*, when extremely abundant, may result in significantly decreased mussel growth and also cause severe problems in crop handling, resulting in increased production costs estimated at $4.5 million [45]. In contrast, several species of ascidians are cultured for food primarily in Japan, Korea and France. The solitary ascidian *Halocynthia roretzi* has long been a popular seafood in Japan and Korea, with a market value of $18 million in 2006 [46]. Recently, a unique infectious agent has been identified as the cause of mass mortality of these cultured ascidians [47].

Ascidians provide a fertile ground for studies in the field of natural products. Similar to sponges and bryozoans, many ascidians avoid predation or fouling by producing noxious secondary metabolites [48]–[52]. Because of these properties, numerous species of ascidians may thus be a potential source of new anti-cancer compounds [53], [54]. Trabectedin (earlier known as ecteinascidin-743, commercial name Yondelis®), a marine-derived alkaloid isolated from extracts of *Ecteinascidia turbinate*, is now being used in treatment of soft-tissue sarcomas [55], [56]. Antimalarial compounds have been isolated from the solitary ascidians *Microcosmus helleri, Ascidia sydneiensis* and *Phallusia nigra* [57], and numerous other compounds with anti-cancer, anti-viral and anti-bacterial capabilities are in various clinical trial stages by the pharmaceutical industry. The management and use of these organisms as sources of natural products is dependent, however, on understanding their taxonomy, the integrative basis of biology.

Ascidians have a poor fossil record [58]. Although calcareous spicules of distinctive shapes are found in some species of the families Polycitoridae, Pyuridae, and especially the Didemnidae [59], [60], their fossils are rarely reported by paleontologists [61]. This is possibly due to their susceptibility to dissolution, and small size; many are less than 0.1 mm [60]. Fossil didemnid ascidian spicules were encountered in rocks from various regions around the world, usually dating to the Late Pliocene-Early Pleistocene period [61], [62]. Eight specimens of a solitary fossil tunicate have been discovered with a body size of 2–4 cm; they resemble the extant *Clavelina* genus and are presumably ∼520 million years old [63].

Currently there are numerous web-based sources of taxonomic inventories (e.g., Encyclopedia of Life http://www.eol.org, Integrated Taxonomic Information System http://www.itis.gov), but only a few websites are dedicated to ascidians (e.g., The Dutch ascidians Home Page http://www.ascidians.com, Ascidian Home Page for United States http://depts.washington.edu/ascidian/), and they do not aim to provide an inventory list. Unfortunately, most web-based datasets often lack updates due to limitations in funding and expertise. The Ascidiacea World Database (http://www.marinespecies.org/ascidiacea/), which is a part of the World Register of Marine Species (WoRMS), is unique; it contains a comprehensive list of ascidian species, including information on synonymy, taxonomic literature, and distribution [64]. This database is the result of a joint effort of several ascidian taxonomists who constantly update and revise the information. With the aid of this database and the large taxonomic literature, our aim is to provide a systematic review of the class Ascidiacea, describe the main regions of highest biodiversity, discuss the risks of invasive species, and summarize the current trends in ascidian global distribution patterns.

## Methods

### Biogeographic distribution

Ascidian specimens are held by museums and similar institutions all over the world. However, only a few institutions provide reliable on-line options to search their collections (e.g., Smithsonian Invertebrate Zoology Collections, The Santa Barbara Museum of Natural History, Yale Peabody Museum Catalog Service, The Online Zoological Collections of Australian Museums). In these on-line collections we were able to find invaluable unpublished information regarding species distribution, and verify the occurrence of certain species in their native or introduced range. In addition, a literature search was done to record the number of species identified in various regions of the world in order to provide an estimate of global species richness. It is important to note that the numbers we provide represent the exact number of species mentioned in each citation.

### Maps and geographic regions

Species distribution information was compiled based on the geographic regions of the Exclusive Economic Zone division v5 standard map provided by VLIZ Maritime Boundaries Geodatabase [65].

### Species names and systematic validation

We followed the taxonomic classification and used the tabular keys of Monniot et al. 1991 [1] (revised by F. Monniot and G. Lambert 2008–2009, unpublished data). Annual check-lists are published on-line by the Catalogue of Life [66], and the Encyclopedia of Life [67]. Both databases are connected to the World Register of Marine Species (WoRMS) check list, to which the Ascidiacea World Database belongs [64]. Therefore, to avoid confusion, only the valid names and classification provided by the Ascidiacea World Database http://www.marinespecies.org/ascidiacea/ were used for systematic analysis of families, genera etc. Division of colonial versus solitary species was based on the Monniot et al. 1991 keys. Taxonomic contribution was analyzed according to the authority index in the World Ascidiacea Database, and only first authors were taken into consideration.

### Records of non-indigenous ascidians

In order to compile a current list of non-indigenous ascidians, we gathered data not only from the available literature but also from different governmental reports which often provide the first record of an introduced species. In addition, valuable information was obtained from the proceedings of the International Invasive Sea Squirt conferences (J Exp Mar Biol Ecol 342 (1), 2007 and Aquat Inv 4 (1) 2009). The list includes only species that are mentioned as introduced or non-indigenous. Species that are classified as “cryptogenic” (species that cannot be reliably demonstrated as being either introduced or native, 68) were not included.

## Results

### Systematic division of ascidian species

**Figure 2:**
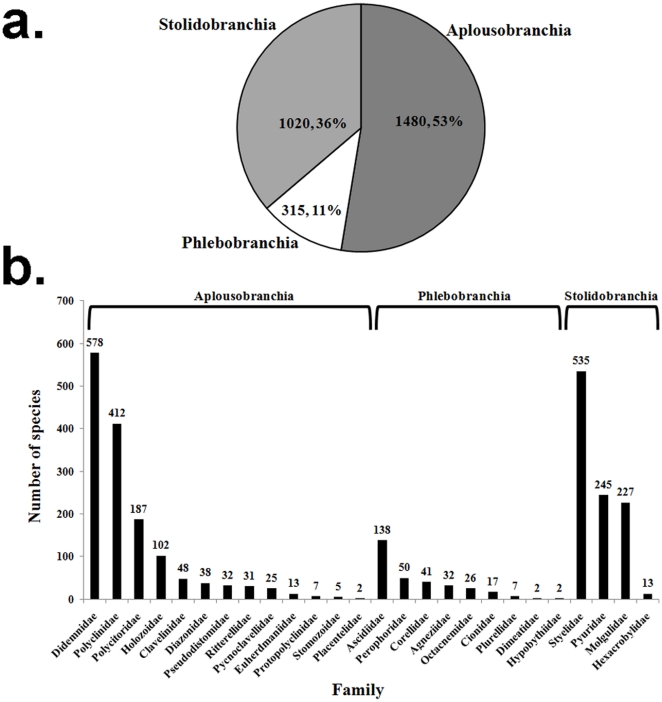
Systematic division of ascidian species. a) Number and percentage of species per order; b) number of species by family within each order.

Our systematic list includes 2815 valid species of ascidians. The highest number of species and families is found in the order Aplousobranchia, with approximately 50% of the species (1480) in the class Ascidiacea (Fig. 2a). Based on the classification of the Ascidiacea World Database, there are currently 26 families in the class Ascidiacea, of which 13 belong to the Aplousobranchia, with the Didemnidae having the highest number of species (578). The genera with the highest number of described species are *Aplidium* (259) and *Didemnum* (228), in the Aplousobranchia. However, the highest number of genera per family was found in the Styelidae (38), order Stolidobranchia, which also has the second highest number of species (535) (Fig. 2b). The majority of described species in the Ascidiacea are colonial (1730, 61.5%) (Table S1, supporting material).

### Discovery rate and author contribution

**Figure 3:**
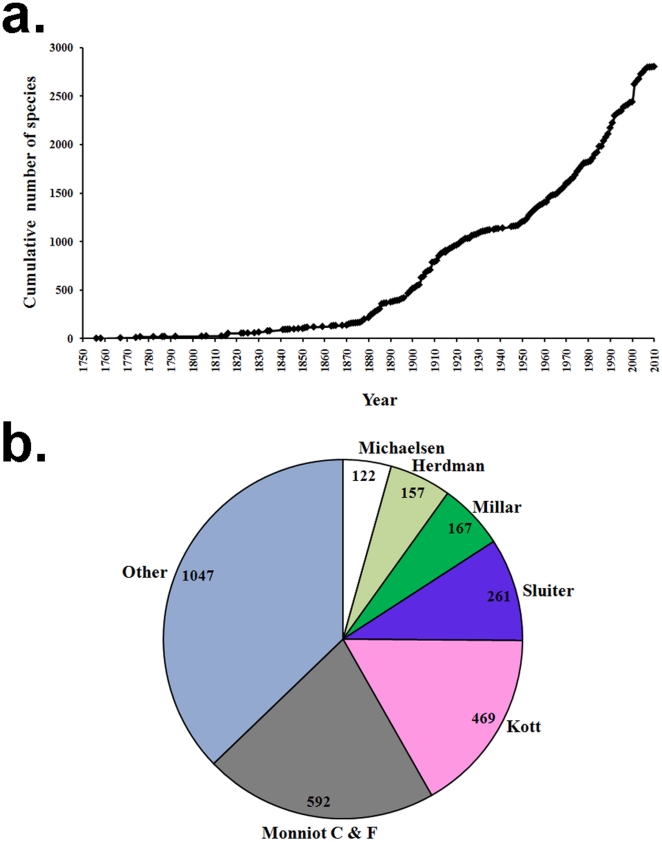
Discovery rate and author contribution. a) Cumulative number of valid ascidian species described between 1750–2010; b) Percentage and number of species described per taxonomic authority. Note: only taxonomic authorities with more than 100 species are mentioned by name.

The discovery rate of ascidian species from 1756 until 2010 is presented in Fig. 3a. The rate of discovery has accelerated since 1950, when the major taxonomists of this group, P. Kott, C. and F. Monniot, and R.H. Millar began publishing. Over 1600 species have been described by these experts including the numerous descriptions by C.P. Sluiter and W.A. Herdman from the late 19^th^ century-beginning of the 20^th^ century. Figure 3b summarizes the contribution of the major taxonomists to total ascidian species described. Only authors responsible for more than 100 descriptions are mentioned by name; Claude and Françoise Monniot were grouped together due to their numerous collaborative publications.

### Non-indigenous ascidians

Review of the literature resulted in records of 64 non-indigenous species (Table 1). Thirty three species are colonial. Half of the introduced species (32) belong to the order Stolidobranchia, the rest divide between the other two orders. Almost half of the species (30) have records only from the northern hemisphere, 13 have records only from the southern hemisphere, and 21 have records from both sections.

**Table 1:** Documented locations of non-indigenous ascidians.

Species	Introduced sites	Lifestyle	Order	Remarks	References
1. *Aplidium glabrum*	Netherlands	C	A	NH	[69]
2. *Aplidium phortax*	New Zealand	C	A	SH	[70]
3. *Aplidium accarense*	Brazil	C	A	SH	[71]
4. *Ascidia archaia*	Atlantic Panama	S	P	T, NH	[72]
5. *Ascidia cannelata*	Mediterranean Sea	S	P	NH	[73]–[75]
6. *Ascidia sp.*	California harbors	S	P	NH	[41], [76]
7. *Ascidia sydneiensis*	Atlantic Panama, Brazil, Guam, Hawaii, India South America	S	P	T	[39], [40], [77]–[82]
8. *Ascidia zara*	California harbors	S	P	NH	[38], [41], [76]
9. *Ascidiella aspersa*	Argentina, New England, New Zealand, South Africa, South Australia, Tasmania	S	P		[82]–[87]
10. *Asterocarpa humilis*	Chile, New Zealand	S	S	SH	[70], [88]
11. *Bostrichobranchus pilularis*	California harbors	S	S	NH	[38]
12. *Botrylloides leachi*	South Australia and Tasmania	C	S	SH	[85], [86]
13. *Botrylloides perspicuum*	California harbors	C	S	NH	[41], [76]
14. *Botrylloides sp.*	New Zealand	C	S	SH	[70]
15. *Botrylloides violaceus*	Alaska, Atlantic Canada, Belgium, California harbors, England, Mediterranean Sea, Netherlands, New England, San Francisco Bay	C	S	NH	[41], [69], [73], [75], [76], [83], [89]–[95]
16. *Botryllus schlosseri*	Atlantic Canada, California harbors, India, New England, San Francisco Bay, South Africa, South Australia and Tasmania, US West coast	C	S		[38], [41], [76], [78], [82], [83], [85], [86], [89], [90], [92], [93], [96]
17. *Ciona intestinalis*	Atlantic Canada, California harbors, Chile, China/Korea, New Zealand, South Africa, South Australia and Tasmania, Washington	S	P		[38], [41], [70], [76], [82], [85]–[89], [97]–[99]
18. *Ciona savignyi*	California harbors, Japana, New Zealand, Washington	S	P		[38], [41], [76], [98], [100], [101]
19. *Clavelina lepadiformis*	NW Atlantic, South Africa	C	A		[82], [102]
20. *Cnemidocarpa areolata (C. irene)*	Brazil	S	S	T, SH	[80]
21. *Cnemidocarpa humilis*	South Africa	S	S	SH	[82]
22. *Cnemidocarpa irene*	Hawaii	S	S	T, NH	[103]
23. *Corella eumyota*	England, Iberia Atlantic coast, New Zealand, NW France	S	P		[70], [91], [104], [105]
24. *Cystodytes philippinensis*	Mediterranean Sea	C	A	NH	[73], [75]
25. *Didemnum cineraceum*	Atlantic Panama, Brazil	C	A	T	[72], [80]
26. *Didemnum perlucidum*	Brazil, Caribbean, Guam, Gulf of Mexico	C	A	T	[40], [71], [106], [107]
27. *Didemnum sp.*	Hawaii	C	A	T, NH	[81]
28. *Didemnum vexillum*	England, New England, San Francisco Bay, Washington, widely distributed	C	A		[83], [90], [98], [108], [109]
29. *Diplosoma listerianum*	Brazil, Guam, Netherlands, New England, South Africa	C	A	T	[40], [69], [71], [82], [83]
30. *Distaplia bermudensis*	Brazil, Florida, Mediterranean Sea	C	A	T	[71], [73], [75], [110]
31. *Distaplia stylifera*	Brazil	C	A	SH	[80]
32. *Ecteinascidia styeloides*	Mediterranean Sea	C	P	NH	[73], [75]
33. *Ecteinascidia thurstoni*	Mediterranean Sea	C	P	NH	[74], [75]
34. *Eudistoma elongatum*	New Zealand	C	A	SH	[111]
35. *Eusynstyela tincta*	India	C	S	NH	[78]
36. *Herdmania momus*	Hawaii, Mediterranean Sea	S	S	T, NH	[39], [73]–[75]
37. *Herdmania pallida*	Atlantic Panama, Hawaii	S	S	T, NH	[72] Lambert unpublished data
38. *Lissoclinum fragile*	Guam	C	A	T, NH	[40]
39. *Microcosmus exasperatus*	Atlantic Panama, Guam, Hawaii, India, Mediterranean Sea	S	S	T, NH	[39], [40], [72]–[74], [75], [78]
40. *Microcosmus squamiger*	California harbors, India, Mediterranean Sea, South Africa	S	S	T, NH	[38], [41], [73], [75], [76], [78], [82], [112]
41. *Molgula citrina*	Alaska	S	S	NH	[113]
42. *Molgula ficus*	California harbors, Chila	S	S		[88], [114]
43. *Molgula manhattensis*	California harbors, China/Korea, Europe, NE Pacific, Netherlands, South Australia and Tasmania	S	S		[38], [41], [69], [76], [86], [94], [115]
44. *Perophora japonica*	Atlantic Europe, England, Netherlands, Northern California	C	A	NH	[69], [91], [116], [117]
45. *Perophora multiclathrata*	Mediterranean Sea	C	A		[73], [75]
46. *Phallusia nigra*	Guam, Hawaii, India, Mediterranean Sea	S	P	T, NH	[39], [40], [73]–[75], [78], [81]
47. *Polyandrocarpa anguinea*	Brazil	C	S	T, SH	[80]
48. *Polyandrocarpa sp.*	Hawaii	C	S	T, NH	[39]
49. *Polyandrocarpa zorritensis*	California harbors, Gulf of Mexico, Mediterranean Sea	C	S	T, NH	[38], [41], [73], [75], [76], [107]
50. *Polycarpa aurita*	Hawaii	S	S	T, NH	[103]
51. *Polycarpa spongiabilis*	Brazil	S	S	SH	[71]
52. *Polycarpa tumida*	Brazil	S	S	T, SH	[80]
53. *Polyclinum aurantium*	Brazil	C	A	SH	[77]
54. *Polyclinum constellatum*	Guam, Brazil, Pacific Mexico	C	A	T	[40], [71], [118]
55. *Pyura praeputialis*	Chile	S	S		[88]
56. *Pyura vittata*	Atlantic Panama	S	S	T, NH	[72]
57. *Rhodosoma turcicum*	Mediterranean Sea, Florida	S	P	NH	[74], [75] Lambert unpublished data
58. *Styela canopus*	Atlantic Panama, Brazil, California harbors, Guam, India	S	S	T	[40], [42], [43], [74], [75], [78], [80], [82]
59. *Styela clava*	Atlantic Canada, California harbors, China/Korea, Denmark, England, Germany, Mediterranean Sea, Netherlands, New England, New Zealand, San Francisco Bay, South Australia and Tasmania, England, Washington	S	S		[38], [41], [70], [73], [75], [76], [85], [86], [90], [91], [95], [98], [99], [119]
60. *Styela plicata*	Brazil, California harbors, China/Korea, Gulf of Mexico, South Africa, South Australia and Tasmania	S	S	T	[38], [41], [76], [77], [79], [82], [85], [86], [99], [107], [120]
61. *Symplegma brakenhielmi*	California harbors, Guam. Hawaii, Mediterranean Sea	C	S	T, NH	[38]–[40], [73]–[75]
62. *Symplegma reptans*	California harbors, Hawaii	C	S	T, NH	[38], [39], [41], [76]
63.*Symplegma rubra*	Brazil, Gulf of Mexico	C	S	T	[71], [107]
64. *Trididemnum* cf. *savignii*	Mediterranean Sea	C	A	NH	[75]

C- Colonial, S- Solitary, Order: A-Aplousobranchia, P-Phlebobranchia, S- Stolidobranchia, Remarks: T- Tropical, NH- Northern Hemisphere only, SH- Southern Hemisphere only.

Records of introduction of ascidians in tropical waters are mainly from Hawaii, Guam and Panama. Of the 64 documented global non-indigenous species, 27 species have records in tropical regions. However, only 14 have records that are restricted to tropical environments (*Ascidia archaia*, *Cnemidocarpa areolata*, *Cnemidocarpa irene, Didemnum cineraceum*, *Didemnum* sp. (Hawaii), *Distaplia stylifera, Herdmania pallida, Lissoclinum fragile, Polyandrocarpa anguinea, Polyandrocarpa* sp., *Polycarpa aurita, Polycarpa tumida, Polyclinum constellatum, Pyura vittata*). The majority of species (50) have introduction records from temperate environments, including both the northern and southern hemispheres. There are no records yet of non-indigenous ascidians from the arctic.

### Ascidian global distribution

**Figure 4:**
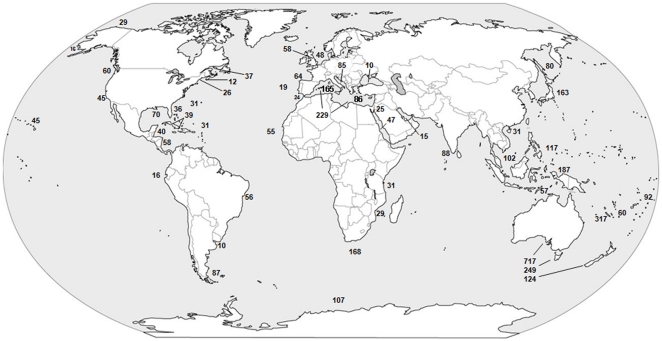
Ascidian global distribution (abyssal species not included).

Fig. 4 provides a summary of species richness in different regions of the world. A complete list of sites and references is provided in Table 2. The highest number of ascidian species is found in the Indo-Pacific, with inventory numbers such as 317 species from New-Caledonia, 187 species from the Western Pacific ocean, 117 and 102 species from Guam and Gulf of Siam area (numbers represent the exact number of species mentioned in each citation). The ascidian fauna along the coasts of Australia and New Zealand was studied extensively, resulting in records of 717 species from Australia, 249 species from Tasmania, and 124 species from New Zealand. At higher latitudes, the Mediterranean and Japan each represent areas with high number of species with about 229 species from the Mediterranean and 163 species in Japan. Antarctica and South Africa also have extensive records of ascidian species of 107 and 168 species respectively. The North American coasts have been studied thoroughly with approximately 170 species along both the Atlantic and Pacific coasts.

**Table 2:** Ascidian regional species richness.

Area	Number of species	Reference
Australia	717	[121]
New Caledonia	317	[122]
Tasmania	249	[123]
Mediterranean Sea	229	[124]
Western Pacific Ocean: Palau, The Philippines, Indonesia, and Papua New Guinea	187	[125]
South Africa	168	[126]
Western Mediterranean	165	[127]
Japan	163	[128]
New Zealand	124	[123]
Guam	117	[129]
Antarctica	107	[32]
Indo West Pacific region	102	[130]
French Polynesia	92	[131]
India	88	[132]
South America	87	[133]
Eastern Mediterranean	86	[127]
Adriatic	85	[127]
North West Pacific (Kamchatka)	80	[134]–[140]
Gulf of Mexico	70	[107]
Gibraltar	66	[141]
Iberia	64	[142]
Fiji	60	[143]
Pacific Northwest	60	[144]
Panama	58	[145]
British	58	[146]
Timor and Arafura Sea	57	[147]
Brazil	56	[148]
Africa north west coast	55	[149]
Chile	55	[150]
Scandinavia	48	[151]
Red Sea	47	[152]
Hawaii	45	[153]
California	45	[154]
Belize	40	[31]
Jamaica	39	[155]
Cuba	39	[156]
Gulf of Saint Lawrence	37	[157]
Florida	36	[154]
Hong Kong	31	[158]
Bermuda	31	[159]
West indies	31	[160]
Tanzania	31	[161]
Mozambique	29	[162], [163]
Circumpolar	29	[113]
Venezuela	29	[164]
Massachusetts	26	[154]
Gulf of Aqaba	25	[165], [166]
Azores Islands	19	[167]
Galapagos	16	[168]
Bering Sea	16	[154]
Bahrain	15	[169]
Bay of Fundy	12	[154]
Black Sea	10	[127]

Data sorted by number of species.

## Discussion

Even though the class Ascidiacea has been the object of much scientific interest in the last decade [170], there are extensive regions around the world where very little collecting of ascidians has been done, resulting in very low number of described ascidian species and general lack of data (e.g., South and Central America, Canada, Alaska, and many thousands of islands in the tropical west Pacific). The current study reveals a strong association between species richness and sampling efforts. In addition, there is a clear trend of arrival and spread of non-indigenous species that put the endemic fauna at risk. Both of these issues emphasize the need for additional research in the field of ascidian biodiversity and biogeography.

In geographical areas where taxonomists have long been active, we typically found high numbers of species. The majority of the described ascidian species (more than 60%) are attributed to only seven taxonomic experts. This is demonstrated in the high species richness found in Australia [121], New Caledonia [122], Japan [128], the Caribbean Sea [171], and South Africa [172]–[174]. In contrast, along the coasts of South America, Indian Ocean, and Eastern Atlantic, there are vast areas with only scarce information regarding the occurrence of ascidians, and in some cases the only information comes from studies that may be out of date and not representative of the diversity these areas currently exhibit [154], [175]. For instance, the ascidian fauna of the Western Mediterranean has been studied in great detail and has been recorded in number of publications with an estimate of 165 described species [127]. The nearby Red Sea, which supports one of the most diverse ecosystems in the world [176] is represented by only 47 described species [152]. Thus, this discrepancy appears to be a result of less research and fewer sampling efforts, rather than a decrease in ascidian diversity [177]. Our inventory of 2815 described species of ascidians is certainly incomplete, with an estimation that approximately 3000 species remain to be discovered and described (Appeltans et al. 2011 unpublished data). Applying molecular approaches may further assist in locating cryptic speciation of a single species.

The high diversity of some of the ascidian families is remarkable. With approximately 26 families in the class Ascidiacea, the colonial Didemnidae family contains 20% of the described species, possibly due to highly diverse *Didemnum* genera, with more than 200 species. The Styelidae family is also highly diverse with 38 genera, and 535 described species, colonial and solitary. Colonial species characterize more than 60% of the described species. The high diversity of colonial ascidians is increasingly important since many contain very active secondary metabolites important to the pharmaceutical industry [178].

In general, it has been shown that in tropical environments colonial species dominate the substrate [179]. This is attributed to their asexual reproduction and indeterminate growth which provide them with a significant advantage for the exploitation of tropical habitats. Thus there are many more colonial ascidian species than solitary species in the tropics, representing about 80% of the species [125], [126], [131], [143], [180]. Although colonial ascidians are generally considered a minor benthic component on exposed surfaces of the natural coral reefs they can rapidly overgrow corals and outcompete them for space during periods of nutrient enrichment [181]–[183]. Since ascidians are able to filter even minute particulate matter [184], [185], any rise in nutrient levels and organic material in coastal waters will have a direct influence on their abundance.

In temperate waters solitary ascidians comprise 52% of the American fauna [154], and 75% in European waters (but this includes abyssal forms, almost all of which are solitary), [125], [186]. In the Antarctic, 58% of the species are solitary [32]. It is possible that solitary ascidians in the Antarctic and the deep sea, many of which are stalked, have an advantage over encrusting colonial species since most of the benthos is composed of soft sediments, so their vertical growth lifts them above the sediment. This three dimensional structure may improve food capture and assimilation during periods of winter inactivity and sedimentation [187]. In addition, since in solitary ascidians fertilization and larval development usually occur in the water column (in contrast to colonial species which are brooders), it is possible that they have a higher potential for dispersal [74]. This may also be advantageous in the Antarctic in cases of anchor ice formation [188], and ice scouring [189] which have a key role in determining marine biodiversity in high latitudes, emphasizing the importance of larval dispersal processes.

Historical baselines for comparison to present day from museum collections and published literature are required in order to understand and respond to changes in global biodiversity [190]. The current study provides a list of 64 non-indigenous ascidians (NIAs) with published records of introduction. This number is likely to be an underestimate, due to difficulty in taxonomic identification of aplousobranch species in particular. In some cases it may be difficult to determine if a certain species record is of a new introduction, or of a previously undiscovered natural population [113]. Lambert [40] suggests two criteria for the designation of NIAs in Guam, following the general guidelines of Chapman and Carlton [68] for determining non-indigenous species: (1) restricted to artificial surfaces and (2) an extra Indo-West Pacific distribution. The first criterion may be especially important especially in tropical environments which may be more resistant to invasion due to their diverse communities [191], [192]. In temperate and cold water environments there are records of rapid spread of NIAs on natural substrates such as *Didemnum vexillum* (Gulf of Maine) [109], [193] and *Microcosmus squamiger* (Western Mediterranean) [112]. A molecular approach, therefore, may be more relevant in revealing the status of a certain species [194]–[196].

The majority of records of NIAs are from cold water environments, suggesting this environment may be more favorable to introductions of ascidians. Nonetheless, nearly half of the NIAs have geographical records from tropical environments. Under lab conditions, at high temperature, the growth rate of NIAs was higher compared to that of native species [197], and they were able to tolerate significantly higher temperatures [198]. Thus, there is growing evidence that global warming may facilitate a shift northward by non-native species, accelerating homogenization of the global biota [199]. Nevertheless, high rates of endemism can be found in tropical environments such as the Great Barrier Reef [121], New Caledonia and French Polynesia [122], [131], and also in unique environments such as Southern New Zealand [123], and the Antarctic, with its isolated and homogeneous fauna [32].

The class Ascidiacea presents vast opportunities for research in the fields of evolution and development, physiology, natural products, and marine bioinvasion. Yet, there are many areas around the world that are relatively poorly known, and in others the available data should be updated and revised. Many more species are yet to be discovered, contributing to our accumulating knowledge of this unique group.

## Supporting Information

Table S1 Systematic division of ascidian species following the Ascidiacea World Database [67].
(DOC)
