# Supplementary material for: Global Diversity of Ascidiacea
Source: PLoS One. 2011 Jun 20;6(6):e20657. doi: 10.1371/journal.pone.0020657 (PMC3119061; doi:10.1371/journal.pone.0020657)
Supplement: Table S1 — Systematic division of ascidian species following the Ascidiacea World Database [67]. (DOC) [file pone.0020657.s001.doc]

| **Supporting material**  Table S1. Systematic division of ascidian species following the Ascidiacea World Database [67].   | **Order** | **Family** | **Genus** | **no. of species per genus** | **Colonial/Solitary** | | --- | --- | --- | --- | --- | | Aplousobranchia | Clavelinidae | Clavelina | 47 | Colonial | |  |  | Nephtheis | 1 | Colonial | |  | Diazonidae | Aphanibranchion | 1 | Colonial | |  |  | Diazona | 12 | Colonial | |  |  | Pseudodiazona | 2 | Colonial | |  |  | Pseudorhopalaea | 1 | Colonial | |  |  | Rhopalaea | 18 | Colonial | |  |  | Syndiazona | 1 | Colonial | |  |  | Tylobranchion | 3 | Colonial | |  | Didemnidae | Atriolum | 9 | Colonial | |  |  | Clitella | 1 | Colonial | |  |  | Coelocormus | 1 | Colonial | |  |  | Didemnum | 228 | Colonial | |  |  | Diplosoma | 41 | Colonial | |  |  | Leptoclinides | 75 | Colonial | |  |  | Lissoclinum | 61 | Colonial | |  |  | Polysyncraton | 94 | Colonial | |  |  | Trididemnum | 68 | Colonial | |  | Euherdmaniidae | Euherdmania | 13 | Colonial | |  | Holozoidae | Distaplia | 54 | Colonial | |  |  | Hypodistoma | 4 | Colonial | |  |  | Hypsistozoa | 3 | Colonial | |  |  | Neodistoma | 1 | Colonial | |  |  | Polydistoma | 4 | Colonial | |  |  | Protoholozoa | 6 | Colonial | |  |  | Pseudoplacentela | 1 | Colonial | |  |  | Sigillina | 13 | Colonial | |  |  | Sycozoa | 16 | Colonial | |  | Placentelidae | Placentela | 2 | Colonial | |  | Polycitoridae | Archidistoma | 4 | Colonial | |  |  | Brevicollus | 1 | Colonial | |  |  | Cystodytes | 21 | Colonial | |  |  | Eucoelium | 8 | Colonial | |  |  | Eudistoma | 121 | Colonial | |  |  | Exostoma | 1 | Colonial | |  |  | Millarus | 1 | Colonial | |  |  | Polycitor | 29 | Colonial | |  |  | Rhombifera | 1 | Colonial | |  | Polyclinidae | Aplidiopsis | 20 | Colonial | |  |  | Aplidium | 259 | Colonial | |  |  | Homoeodistoma | 1 | Colonial | |  |  | Macrenteron | 1 | Colonial | |  |  | Morchellium | 6 | Colonial | |  |  | Neodictyon | 1 | Colonial | |  |  | Polyclinella | 1 | Colonial | |  |  | Polyclinum | 42 | Colonial | |  |  | Sidneioides | 4 | Colonial | |  |  | Synoicum | 77 | Colonial | |  | Protopolyclinidae | Condominium | 1 | Colonial | |  |  | Monniotus | 5 | Colonial | |  |  | Protopolyclinum | 1 | Colonial | |  | Pseudodistomidae | Anadistoma | 1 | Colonial | |  |  | Citorclinum | 1 | Colonial | |  |  | Pseudodistoma | 30 | Colonial | |  | Pycnoclavellidae | Euclavella | 1 | Colonial | |  |  | Pycnoclavella | 24 | Colonial | |  | Ritterellidae | Dumus | 1 | Colonial | |  |  | Pharyngodictyon | 6 | Colonial | |  |  | Ritterella | 24 | Colonial | |  | Stomozoidae | Stomozoa | 5 | Colonial | | Phlebobranchia | Agneziidae | Adagnesia | 12 | Solitary | |  |  | Agnezia | 13 | Solitary | |  |  | Caenagnesia | 3 | Solitary | |  |  | Proagnesia | 1 | Solitary | |  |  | Pterygascidia | 3 | Solitary | |  | Ascidiidae | Ascidia | 116 | Solitary | |  |  | Ascidiella | 3 | Solitary | |  |  | Fimbrora | 1 | Solitary | |  |  | Phallusia | 17 | Solitary | |  |  | Psammascidia | 1 | Solitary | |  | Cionidae | Araneum | 2 | Solitary | |  |  | Ciona | 14 | Solitary | |  |  | Tantillulum | 1 | Solitary | |  | Corellidae | Abyssascidia | 3 | Solitary | |  |  | Chelyosoma | 9 | Solitary | |  |  | Clatripes | 1 | Solitary | |  |  | Corella | 9 | Solitary | |  |  | Corelloides | 1 | Solitary | |  |  | Corellopsis | 2 | Solitary | |  |  | Corynascidia | 10 | Solitary | |  |  | Dextrogaster | 1 | Solitary | |  |  | Mysterascidia | 1 | Solitary | |  |  | Rhodosoma | 2 | Solitary | |  |  | Xenobranchion | 2 | Solitary | |  | Dimeatidae | Dimeatus | 2 | Solitary | |  | Hypobythiidae | Hypobythius | 2 | Solitary | |  | Octacnemidae | Benthascidia | 1 | Solitary | |  |  | Cibacapsa | 1 | Solitary | |  |  | Cryptia | 1 | Solitary | |  |  | Dicopia | 3 | Solitary | |  |  | Kaikoja | 2 | Solitary | |  |  | Megalodicopia | 2 | Solitary | |  |  | Myopegma | 1 | Solitary | |  |  | Octacnemus | 6 | Solitary | |  |  | Polyoctacnemus | 1 | Colonial | |  |  | Situla | 8 | Solitary | |  | Perophoridae | Ecteinascidia | 28 | Colonial | |  |  | Perophora | 22 | Colonial | |  | Plurellidae | Microgastra | 1 | Solitary | |  |  | Plurella | 6 | Colonial | | Stolidobranchia | Hexacrobylidae | Asajirus | 8 | Solitary | |  |  | Oligotrema | 5 | Solitary | |  | Molgulidae | Anomopera | 1 | Solitary | |  |  | Bostrichobranchus | 3 | Solitary | |  |  | Eugyra | 30 | Solitary | |  |  | Fungulus | 5 | Solitary | |  |  | Gamaster | 3 | Solitary | |  |  | Minipera | 4 | Solitary | |  |  | Molgula | 142 | Solitary | |  |  | Molguloides | 17 | Solitary | |  |  | Namiella | 1 | Solitary | |  |  | Paramolgula | 4 | Solitary | |  |  | Pareugyrioides | 11 | Solitary | |  |  | Protomolgula | 3 | Solitary | |  |  | Rhizomolgula | 2 | Solitary | |  |  | Xenomolgula | 1 | Solitary | |  | Pyuridae | Bathypera | 5 | Solitary | |  |  | Bathypyura | 2 | Solitary | |  |  | Boltenia | 10 | Solitary | |  |  | Bolteniopsis | 4 | Solitary | |  |  | Claudenus | 1 | Solitary | |  |  | Cratostigma | 7 | Solitary | |  |  | Ctenyura | 4 | Solitary | |  |  | Culeolus | 26 | Solitary | |  |  | Halocynthia | 15 | Solitary | |  |  | Hartmeyeria | 9 | Solitary | |  |  | Hemistyela | 3 | Solitary | |  |  | Herdmania | 17 | Solitary | |  |  | Heterostigma | 6 | Solitary | |  |  | Microcosmus | 38 | Solitary | |  |  | Paraculeolus | 1 | Solitary | |  |  | Pyura | 96 | Solitary | |  |  | Pyurella | 1 | Solitary | |  | Styelidae | Alloeocarpa | 9 | Colonial | |  |  | Ärnbäckia | 1 | Colonial | |  |  | Asterocarpa | 2 | Solitary | |  |  | Bathyoncus | 5 | Solitary | |  |  | Bathystyeloides | 7 | Solitary | |  |  | Berillia | 1 | Colonial | |  |  | Botryllocarpa | 2 | Colonial | |  |  | Botrylloides | 14 | Colonial | |  |  | Botryllus | 33 | Colonial | |  |  | Chorizocarpa | 3 | Colonial | |  |  | Chorizocormus | 1 | Colonial | |  |  | Cnemidocarpa | 82 | Solitary | |  |  | Dendrodoa | 8 | Solitary | |  |  | Dextrocarpa | 2 | Colonial | |  |  | Dicarpa | 12 | Solitary | |  |  | Distomus | 7 | Colonial | |  |  | Eusynstyela | 11 | Colonial | |  |  | Gynandrocarpa | 1 | Colonial | |  |  | Kükenthalia | 1 | Colonial | |  |  | Metandrocarpa | 15 | Colonial | |  |  | Monandrocarpa | 6 | Solitary | |  |  | Oculinaria | 2 | Colonial | |  |  | Oligocarpa | 2 | Colonial | |  |  | Pelonaia | 1 | Solitary | |  |  | Podostyela | 1 | Solitary | |  |  | Polyandrocarpa | 30 | Colonial | |  |  | Polycarpa | 137 | Solitary | |  |  | Polyzoa | 9 | Colonial | |  |  | Protostyela | 2 | Solitary | |  |  | Psammostyela | 1 | Solitary | |  |  | Seriocarpa | 5 | Solitary | |  |  | Stolonica | 28 | Colonial | |  |  | Styela | 72 | Solitary | |  |  | Styelopsis | 1 | Solitary | |  |  | Symplegma | 12 | Colonial | |  |  | Syncarpa | 2 | Colonial | |  |  | Theodorella | 3 | Colonial | |  |  | Tibitin | 4 | Colonial | |  |  | **TOTAL** | 2815 |  | |
| --- | --- | --- | --- | --- | --- | --- | --- | --- | --- | --- | --- | --- | --- | --- | --- | --- | --- | --- | --- | --- | --- | --- | --- | --- | --- | --- | --- | --- | --- | --- | --- | --- | --- | --- | --- | --- | --- | --- | --- | --- | --- | --- | --- | --- | --- | --- | --- | --- | --- | --- | --- | --- | --- | --- | --- | --- | --- | --- | --- | --- | --- | --- | --- | --- | --- | --- | --- | --- | --- | --- | --- | --- | --- | --- | --- | --- | --- | --- | --- | --- | --- | --- | --- | --- | --- | --- | --- | --- | --- | --- | --- | --- | --- | --- | --- | --- | --- | --- | --- | --- | --- | --- | --- | --- | --- | --- | --- | --- | --- | --- | --- | --- | --- | --- | --- | --- | --- | --- | --- | --- | --- | --- | --- | --- | --- | --- | --- | --- | --- | --- | --- | --- | --- | --- | --- | --- | --- | --- | --- | --- | --- | --- | --- | --- | --- | --- | --- | --- | --- | --- | --- | --- | --- | --- | --- | --- | --- | --- | --- | --- | --- | --- | --- | --- | --- | --- | --- | --- | --- | --- | --- | --- | --- | --- | --- | --- | --- | --- | --- | --- | --- | --- | --- | --- | --- | --- | --- | --- | --- | --- | --- | --- | --- | --- | --- | --- | --- | --- | --- | --- | --- | --- | --- | --- | --- | --- | --- | --- | --- | --- | --- | --- | --- | --- | --- | --- | --- | --- | --- | --- | --- | --- | --- | --- | --- | --- | --- | --- | --- | --- | --- | --- | --- | --- | --- | --- | --- | --- | --- | --- | --- | --- | --- | --- | --- | --- | --- | --- | --- | --- | --- | --- | --- | --- | --- | --- | --- | --- | --- | --- | --- | --- | --- | --- | --- | --- | --- | --- | --- | --- | --- | --- | --- | --- | --- | --- | --- | --- | --- | --- | --- | --- | --- | --- | --- | --- | --- | --- | --- | --- | --- | --- | --- | --- | --- | --- | --- | --- | --- | --- | --- | --- | --- | --- | --- | --- | --- | --- | --- | --- | --- | --- | --- | --- | --- | --- | --- | --- | --- | --- | --- | --- | --- | --- | --- | --- | --- | --- | --- | --- | --- | --- | --- | --- | --- | --- | --- | --- | --- | --- | --- | --- | --- | --- | --- | --- | --- | --- | --- | --- | --- | --- | --- | --- | --- | --- | --- | --- | --- | --- | --- | --- | --- | --- | --- | --- | --- | --- | --- | --- | --- | --- | --- | --- | --- | --- | --- | --- | --- | --- | --- | --- | --- | --- | --- | --- | --- | --- | --- | --- | --- | --- | --- | --- | --- | --- | --- | --- | --- | --- | --- | --- | --- | --- | --- | --- | --- | --- | --- | --- | --- | --- | --- | --- | --- | --- | --- | --- | --- | --- | --- | --- | --- | --- | --- | --- | --- | --- | --- | --- | --- | --- | --- | --- | --- | --- | --- | --- | --- | --- | --- | --- | --- | --- | --- | --- | --- | --- | --- | --- | --- | --- | --- | --- | --- | --- | --- | --- | --- | --- | --- | --- | --- | --- | --- | --- | --- | --- | --- | --- | --- | --- | --- | --- | --- | --- | --- | --- | --- | --- | --- | --- | --- | --- | --- | --- | --- | --- | --- | --- | --- | --- | --- | --- | --- | --- | --- | --- | --- | --- | --- | --- | --- | --- | --- | --- | --- | --- | --- | --- | --- | --- | --- | --- | --- | --- | --- | --- | --- | --- | --- | --- | --- | --- | --- | --- | --- | --- | --- | --- | --- | --- | --- | --- | --- | --- | --- | --- | --- | --- | --- | --- | --- | --- | --- | --- | --- | --- | --- | --- | --- | --- | --- | --- | --- | --- | --- | --- | --- | --- | --- | --- | --- | --- | --- | --- | --- | --- | --- | --- | --- | --- | --- | --- | --- | --- | --- | --- | --- | --- | --- | --- | --- | --- | --- | --- | --- | --- | --- | --- | --- | --- | --- | --- | --- | --- | --- | --- | --- | --- | --- | --- | --- | --- | --- | --- | --- | --- | --- | --- | --- | --- | --- | --- | --- | --- | --- | --- | --- | --- | --- | --- | --- | --- | --- | --- | --- | --- | --- | --- | --- | --- | --- | --- | --- | --- | --- | --- | --- | --- | --- | --- | --- | --- | --- | --- | --- | --- | --- | --- | --- | --- | --- | --- | --- | --- | --- | --- | --- | --- | --- | --- | --- | --- | --- | --- | --- | --- | --- | --- | --- | --- | --- | --- | --- | --- | --- | --- | --- | --- | --- | --- | --- | --- | --- | --- | --- | --- | --- | --- | --- | --- | --- | --- | --- | --- | --- | --- | --- | --- | --- | --- | --- | --- | --- | --- | --- | --- | --- | --- | --- | --- | --- | --- | --- | --- | --- | --- | --- | --- | --- | --- | --- | --- | --- | --- | --- | --- | --- | --- | --- | --- | --- | --- | --- | --- | --- | --- | --- | --- | --- | --- | --- | --- | --- | --- | --- | --- | --- | --- | --- | --- | --- | --- | --- | --- | --- | --- | --- | --- | --- | --- | --- | --- | --- | --- | --- | --- | --- | --- | --- | --- | --- | --- | --- | --- | --- | --- | --- | --- | --- | --- | --- | --- | --- | --- | --- | --- | --- | --- | --- | --- | --- | --- | --- | --- | --- | --- | --- | --- | --- | --- | --- | --- | --- | --- | --- | --- | --- | --- | --- | --- | --- | --- | --- | --- | --- | --- | --- | --- | --- | --- | --- | --- | --- | --- | --- | --- | --- | --- | --- | --- | --- | --- | --- | --- | --- | --- | --- | --- | --- | --- | --- | --- | --- | --- | --- | --- | --- | --- | --- | --- | --- | --- | --- | --- | --- | --- | --- | --- | --- | --- | --- | --- | --- |
|  |
|  |
|  |
